# Development and validation of a cardiac surgery-associated acute kidney injury prediction model using the MIMIC-IV database

**DOI:** 10.1371/journal.pone.0325151

**Published:** 2025-06-12

**Authors:** Yang Xu, Chunxiao Song, Wenping Wei, Runfeng Miao

**Affiliations:** 1 Department of Emergency, Affiliated Hospital of Yangzhou University, Yangzhou University, Jiangsu, China; 2 Department of Pediatrics, Affiliated Hospital of Yangzhou University, Yangzhou University, Jiangsu, China; Stanford University School of Medicine, UNITED STATES OF AMERICA

## Abstract

**Objective:**

This study aimed to develop an innovative early prediction model for acute kidney injury (AKI) following cardiac surgery in intensive care unit (ICU) settings, leveraging preoperative and postoperative clinical variables, and to identify key risk factors associated with AKI.

**Methods:**

Retrospective data from 1,304 cardiac surgery patients (1,028 AKI cases and 276 non-AKI controls) were extracted from the MIMIC-IV database. We analyzed three datasets: preoperative 48-hour averages, preoperative 48-hour maxima, and postoperative 24-hour maxima of critical physiological parameters. Using logistic regression, LASSO regression, and random forest (RF) algorithms, we constructed nine prediction models, evaluating their performance via AUROC, sensitivity, specificity, Youden’s index, decision curve analysis (DCA), and calibration curves.

**Results:**

Our findings demonstrate that all models achieved AUROC values >0.7, with three models exceeding 0.75. Notably, the logistic regression model incorporating preoperative 48-hour maximum values and postoperative 24-hour maximum values exhibited the highest predictive accuracy (AUROC = 0.755, 95%CI: 0.7185–0.7912), outperforming other configurations. This model’s superiority lies in its integration of dynamic preoperative and postoperative variables, capturing both baseline risks and acute postoperative changes. By systematically comparing multiple machine learning approaches, our study highlights the utility of combining temporal physiological metrics to enhance AKI risk stratification. These results offer a robust, clinically applicable tool for early AKI prediction, enabling proactive interventions to improve outcomes in cardiac surgery patients.

## Introduction

Acute kidney injury (AKI) is a common complication of cardiac surgery, and the incidence of cardiac surgery–associated acute kidney injury (CSA-AKI) is as high as 20% to 30% [1]. At least approximately 3% of CSA-AKI patients require temporary renal replacement therapy (RRT). The perioperative mortality rate for patients with severe AK is three to eight times greater than that for patients without AKI, resulting in longer ICU admissions and hospital stays and increasing the cost of medical care during hospitalization [2]. Patients who undergo cardiac surgery have an increased incidence of acute kidney injury and are four times more likely to die in the hospital [3]. Therefore, we need a clinical prediction model that has some predictive power and is relatively easy to obtain, which can also play an important role in clinicians’ decision-making about treatment options.

The pathophysiology of CSA-AKI is multifactorial, and the exact pathophysiological mechanisms are not fully understood. The major pathways that may be involved include insufficient renal perfusion, ischemia‒reperfusion injury (IRI), inflammatory cascade activation, oxidative stress, nephrotoxin exposure, and genetic polymorphisms, which may occur at any time in the perioperative period [4]. Each of these factors interacts before, during, and after surgery [5]. Therefore, we believe that the changes in vital signs of surgical patients before, during, and after surgery, as well as the results of various laboratory tests, can be used as potential predictors of CSA-AKI.

Generally, before elective surgery, a number of routine preoperative laboratory tests are completed in most patients in order to assess the patient’s suitability for surgery. Based on the available literature, these tests can be categorized as random, indicated, routine, or screening tests. Routine laboratory tests include complete blood count (CBC), basal metabolic panel (BMP) and coagulation tests, such as the partial thromboplastin time (PTT), prothrombin time (PT), international normalized ratio (INR), and blood grouping and antibody screening (T&S) [6]; these tests are used for the detection of unsuspected diseases [4]. Considering the easy accessibility of these laboratory indicators, in this study, we utilized large public databases for screening routine laboratory indicators in parallel retrospective studies.

By reading the articles related to the MIMIC database, it can be determined that there is a lack of clear criteria for the extraction of inspection result data in the database in previous articles; in Fangqi Hu et al. the first inspection data was extracted [7]; in Wei Jiang et al. the average value of the inspection data was extracted due to the consideration that multiple variables were measured more than once [8]; while in our study the maximum value was extracted based on the addition of the maximum value. In our study, in addition to the above two extraction methods, we additionally extracted the maximum value. This retrospective study used the MIMIC-IV database and focused on patients admitted to the ICU after cardiac major vascular surgery. Multiple predictive models were developed using logistic regression, LASSO regression, and random forest regression based on the results of several routine laboratory tests in critical care laboratory patients. And, we will compare the predictive performance of each predictive model using area under the ROC curve (AUROC), decision curve analysis (DCA), and calibration curve analysis to validate the predictive value of predictive models for the development of AKI in ICU patients after cardiac surgery, and obtain the best model.

## Materials and methods

### Data acquisition

The data for this study were obtained from the Critical Care Medical Information Market Database version 2.2 (MIMIC-IV v2.2). MIMIC-IV is a publicly accessible critical care data repository from a single medical centre that collaborates with Beth Israel Deaconess Medical Center (BIDMC, Boston, MA, USA) and the Massachusetts Institute of Technology Laboratory of Computational Physiology (MIT, Cambridge, MA, USA). It is divided into “modules” to reflect the source of the data [9].

The database includes data on more than 257,000 different patients treated at Beth Israel Deaconess Medical Center (BIDMC) in Boston, Massachusetts, between 2008 and 2019, 524,000 of whom were admitted. It also contains the comprehensive records of 73,181 patients hospitalized in various intensive care units [10]. The dataset includes demographic indicators, vital sign readings, laboratory results, fluid balance assessments, and patient survival data, in addition to International Classification and Revision of Diseases (ICD-9 and ICD-10) codes, which provide a standardized framework for systematic classification. In our study, the researcher responsible for extracting the data had access to the database [10].

### Study cohort selection

We screened patients in the MIMIC-IV database who had undergone coronary artery bypass grafting (CABG), valve replacement or repair, or combined CABG and valve surgery using the International Classification of Diseases, Ninth Edition (ICD-9, code 361%) and Tenth Edition (ICD10, code 021%). For patients who underwent multiple major cardiac vascular surgeries, we only included information on the first surgery, and for patients who had multiple ICU admissions after major cardiac vascular surgery, we only considered information on the first ICU admission.

Secondary eligibility assessment of screened cardiac surgery patients was then performed by applying the following exclusion criteria: patients under the age of 18 years, patients who were not admitted to the ICU after cardiac surgery, patients who had an ICU stay of less than 24 hours, patients with missing information on laboratory bioindicators, and patients for whom postoperative AKI status could not be ascertained. A total of 1,304 patients who met the inclusion criteria were ultimately included, including 1,028 AKI patients and 276 non-AKI patients.

### Variable selection

We extracted information using Structured Query Language (SQL) in PostgreSQL (version 13.7.2) and Navicate Premium (version 16) software. The extracted latent variables can be categorized into the following five main groups: 1) demographic data, including age, weight, height, BMI, sex, Sepsis-related Organ Failure Assessment (SOFA) score and Glasgow Coma Scale (GCS); 2) vital signs, including heart rate, respiration, temperature, systolic blood pressure, diastolic blood pressure, mean arterial blood pressure, and oxygen saturation; 3) comorbidities, including obesity, diabetes, hypertension, atrial fibrillation, myocardial infarction, chronic lung disease, chronic renal failure, liver disease, shock, hyperlipidaemia, and vascular disease; 4) laboratory indices, including white blood cell count, red blood cell count, mean erythrocyte haemoglobin content, mean erythrocyte haemoglobin concentration, platelet count, haemoglobin volume, international normalized ratio (INR), plasminogen time, activated partial thromboplastin time, alanine aminotransferase, methionine aminotransferase, alkaline phosphatase, total bilirubin, urea, and creatinine in the first 48 hours of the procedure; blood chloride, blood potassium, blood sodium, blood glucose, white blood cell count, lymphocyte count, monocyte count, neutrophil count, erythrocyte count, mean erythrocyte haemoglobin content, mean erythrocyte haemoglobin concentration, platelet count, haemoglobin volume, mean erythrocyte volume, platelet count, international normalized ratio, prothrombin time, and activated partial thromboplastin time in the first 24 hours of the operation, partial pressure of oxygen, partial pressure of carbon dioxide, oxygenation index, pH, lactate, urea, creatinine, blood calcium, blood chloride, and blood potassium; 5) outcome indicators: the occurrence of AKI, the number of days of ICU stay, and in-hospital mortality. The primary outcome variable of this study was whether AKI occurred after cardiac surgery, and the secondary outcome variables were the length of ICU stay and in-hospital mortality.

It should be noted that in this study, we extracted three different sets of data from the mimic-iv database: 1) the first set of data was included for the first laboratory variables in the patient’s preoperative 48 hours and postoperative 24 hours as group Q1; 2) considering that multiple variables were measured more than once, the second set of data was included for the patient’s preoperative 48 hours and postoperative 24 hours laboratory variables were averaged as group Q2; 3) and the third set of data incorporated was the maximum value of the patient’s laboratory variables during the 48 hours preoperative and 24 hours postoperative as group Q3.

### Data analysis

First, we applied R language version 4.3.2 to randomly assign the research cohort to the training group (70%) and the validation group (30%). Then, we built prediction models based on the training cohort and finally validated them in the validation cohort. Because missing data are very common in the MIMIC database, a potential variable was not included in the analysis in this study if it had a missing data rate greater than 20%. We used SPSS 27.0 to supplement missing data by multiple interpolation for latent variables with missing data rates less than or equal to 20%.

Comparisons between groups were made using the Mann-Whitney U test, Fisher exact test, or Χ2 test, as appropriate.

Data were combined and then screened and analyzed using Stata 18.0. Non-normally distributed continuous variables were expressed as median (interquartile spacing), normally distributed continuous variables as mean ± standard deviation, and categorical variables as percentages.

First, due to the large number of extracted variables, we first used SPSS 27. 0 to perform one-way analysis of variance with P < 0.05 as statistically significant, and initially screened out potential variables with significant differences in order to avoid overfitting and improve the fit of the model. Non-normal continuous variables were tested using the Mann-Whitney U test (non-parametric U test) test and categorical variables using the χ2 test.

Then, we used logistic regression analysis (spss27.0), lasso regression analysis (r-language version 4.3.2) and random forest (r-language version 4.3.2) to further compute and analyze the screened latent variables to derive the risk factors and coefficients, and we built a risk prediction model based on the results of this computation.

Finally, considering that the prediction discriminations of all models were evaluated using consistency statistics, we calculated the AUC value, DCA decision curve, accuracy, sensitivity, and Yoden index of each prediction model separately, as well as calculated the Integrated Discriminant Improvement Index (IDI), and selected the best prediction model by comparing the above consistency statistics.

We plotted the calibration curves of the predictive models using the validation group and assessed the calibration ability of the predictive models by internal validation. The nomograms were plotted according to the weights of the variables in the best predictive model for clinical use.

## Results

**Fig 1:**
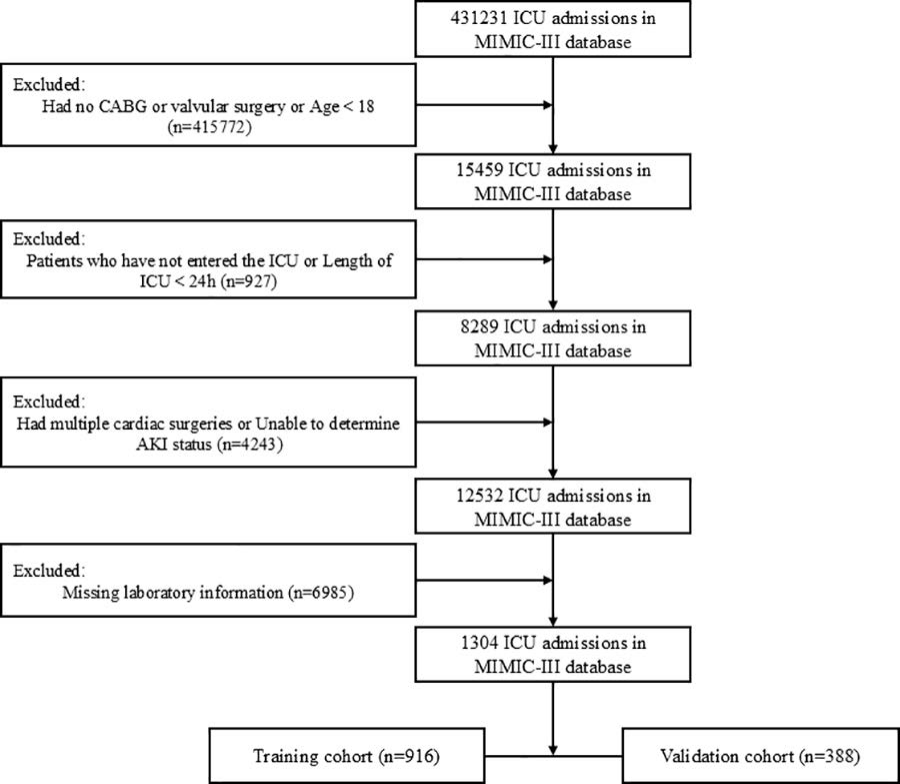
Flowchart of cohort screening. ICU, intensive care unit; MIMIC-IV, Medical Information Mart for Intensive Care IV; CABG, coronary artery bypass grafting; AKI, acute kidney injury.

The number of patients in the MIMIC IV database who underwent elective coronary artery bypass grafting (CABG), valve surgery, or both totaled 15,459, of which a total of 12,532 were admitted to the ICU. We screened these patients by applying the screening criteria of this study, and a total of 1304 patients were included (Fig 1).

### Patient baseline characteristics

The detailed characteristics of all included patients are listed in Table 1. The training cohort consisted of 916 patients, and the validation cohort consisted of 388 patients. The comparison of the median and quartiles of preoperative vital signs, preoperative and postoperative laboratory indices of patients in the training cohort and the validation cohort, as well as the percentage of patients with multiple complications among the total number of patients, revealed little variability. No statistically significant difference was noted between the training and validation cohorts (S1 Table).

**Table 1:** Characterization of patients in the training and validation cohort at baseline.

Categories	Q1 (n = 1304)		Q2 (n = 1304)		Q3 (n = 1304)	
	Modeling group (n = 916)	Vadidation group (n = 388)	Modeling group (n = 916)	Vadidation group (n = 388)	Modeling group (n = 916)	Vadidation group (n = 388)
Age (years)	69.8 (61.9,77.1)	69.7 (61.4,77.5)	69.8 (61.9,77.0)	69.7 (61.4,77.5)	69.8 (61.9,77.0)	69.7 (61.4,77.5)
Weight (kg)	85.4 (73.8,97.4)	86.6 (73.8,99.0)	85.4 (73.9,97.4)	86.6 (73.8,99.0)	85.4 (73.9,97.4)	856.6 (73.8,99.0)
Height (cm)	173.0 (165.0,178.0)	173.0 (165.0,178.0)	173.0 (165.0,178.0)	173.0 (165.0,178.0)	173.0 (165.0,178.0)	173.0 (165.0,178.0)
BMI (kg/m2)	29.2 (25.7,33.0)	29.6 (25.8,33.9)	29.3 (25.7,33.0)	29.6 (25.9,33.9)	29.2 (25.7,33.0)	29.6 (25.8,33.9)
Sex (Male,%)	69.3	73.5	69.3	73.5	69.3	73.5
SOFA	4.0 (2.0,5.0)	4.0 (2.0,5.0)	4.0 (2.0,5.0)	4.0 (2.0,5.0)	4.0 (2.0,5.0)	4.0 (2.0,5.0)
GCS	15.0 (14.0,15.0)	15.0 (14.0,15.0)	15.0 (14.0,15.0)	15.0 (14.0,15.0)	15.0 (14.0,15.0)	15.0 (14.0,15.0)
Vital signs						
Heart rate (times/min)	79.3 (73.7,85.2)	79.0 (73.7,83.9)	79.3 (73.7,85.2)	79.0 (73.7,83.9)	79.3 (73.7,85.2)	79.0 (73.8,83.9)
Respiratory (times/min)	17.0 (15.6,18.6)	17.2 (15.8,18.6)	17.0 (15.6,18.6)	17.2 (15.8,18.6)	17.0 (15.6,18.6)	17.2 (15.8,18.6)
Temperature (°C)	36.5 (36.3,36.7)	36.5, (36.3,36.7)	36.5 (36.3,36.7)	36.5, (36.3,36.7)	36.5 (36.3,36.7)	36.5, (36.3,36.7)
Systolic blood pressure (mmHg)	112.0 (106.5,117.6)	111.6 (106.6,118.4)	111.9 (106.5,117.5)	111.6 (106.1,118.5)	111.9 (106.5,117.6)	111.6 (106.0,118.3)
Diastolic blood pressure (mmHg)	57.5 (52.6,62.0)	57.6 (52.7,62.4)	57.3 ± 7.0	57.2 ± 7.2	57.3 ± 7.0	57.2 ± 7.3
Mean arterial pressure (mmHg)	75.4 (71.2,79.7)	75.0 (71.1,79.5)	75.3 (71.2,79.6)	75.0 (71.1,79.5)	75.3 (71.2,79.6)	75.0 (71.1,79.5)
Oxygen saturation (%)	98.5 (97.5,99.3)	98.5 (97.5,99.2)	98.5 (97.6,99.3)	98.4 (97.5,99.2)	98.5 (97.5,99.3)	98.5 (97.5,99.2)
Commorbidities (%)						
Obesity	17.2	17.3	17.2	17.3	17.2	17.3
Diabetes	36.2	35.6	36.2	35.6	36.2	35.6
Hypertension	53.7	50.8	53.7	50.8	53.7	50.8
Atrial fibrillation	45.5	47.2	45.5	47.2	45.5	47.2
Myocardial infarction	28.3	30.2	28.3	30.2	28.3	30.2
Heart failure	14.6	13.1	14.6	13.1	14.6	13.1
Chronic lung disease	3.9	8.2	3.9	8.2	3.9	8.2
CKD	15.9	17.8	15.9	17.8	15.9	17.8
Liver disease	0.7	0.8	0.7	0.8	0.7	0.8
Stroke	0.7	0.8	0.7	0.8	0.7	0.8
Hyperlipidemia	66.7	68.6	66.7	68.6	66.7	68.6
Vascular disease	4.7	4.1	4.7	4.1	4.7	4.1
Preoperative laboratory indicators (Unit)						
WBC (*109/L)	7.3 (5.9,8.7)	7.15 (5.8,8.8)	7.3 (5.9,8.7)	7.2 (5.8,8.7)	7.4 (6.0,8.8)	7.3 (6.0,8.8)
RBC (*109/L)	4.2 (3.8,4.6)	4.2 (3.8,4.7)	4.2 (3.8,4.6)	4.2 (3.8,4.7)	4.2 (3.8,4.6)	4.3 (3.8,4.7)
HB (g/dL)	12.9 (11.5,13.9)	12.9 (11.5,14.1)	12.8 (11.4,13.9)	30.0 (28.8,31.4)	12.9 (11.5,14.0)	12.9 (11.5,14.2)
MCH (pg)	30.3 (29.0,31.6)	30.0 (28.9,31.5)	30.3 (29.0,31.5)	30.0 (28.8,31.4)	30.4 (29.0,31.6)	30.1 (28.9,31.6)
MCHC (%)	33.2 (32.3,34.1)	33.1 (32.3,33.9)	33.2 (32.4,34.0)	33.1 (32.2,33.9)	33.3 (32.5,31.6)	33.2 (32.4,34.0)
INR (s)	1.1 (1.0,1.2)	1.1 (1.0,1.2)	11.1 (1.0,1.2)	1.1 (1.0,1.2)	1.1 (1.0,1.2)	1.1 (1.0,1.2)
PT (s)	12.0 (11.3,12.9)	12.0 (11.2,13.0)	12.0 (11.3,12.9)	12.0 (11.3,13.0)	12.1 (11.3,13.0)	12.0 (11.3,13.1)
ALP (IU/L)	71.0 (56.0,89.0)	71.0 (58.2,92.0)	70.0 (56.0,89.0)	71.6 (59.0,91.0)	71.0 (56.0,89.0)	72.0 (59.0,91.0)
BUN (mg/dL)	18.8 (15.0,24.0)	18.5 (15.0,24.0)	18.0 (15.0,23.8)	18.7 (15.0,24.0)	19.0 (15.0,24.0)	19.0 (15.0,25.0)
Creatinine (mg/dL)	1.0 (0.8,1.2)	1.0 (0.8,1.2)	1.0 (0.8,1.2)	1.0 (0.8,1.2)	1.0 (0.8,1.2)	1.0 (0.9,1.2)
Blood glucose (mg/dL)	111.0 (96.2,143.0)	110.5 (96.0,138.5)	113.0 (97.0,145.0)	111.0 (97.0,138.7)	117.0 (99.0,151.0)	114.0 (97.2,141.7)
Postoperative laboratory indicators (Unit)						
WBC (*109/L)	9.9 (7.3,13.5)	10.0 (7.2,13.8)	12.5 (9.3,16.4)	12.4 (9.3,16.5)	15.1 (11.4,19.4)	15.0 (11.6,20.0)
MO (*109/L)	0.42 (0.25,0.62)	0.41 (0.27,0.62)	0.41 (0.24,0.61)	0.43 (0.27,0.60)	0.42 (0.24,0.61)	0.4 (0.2,0.6)
NEUT (*109/L)	9.3 (6.5,12.3)	8.9 (6.4,12.4)	9.2 (6.4,12.0)	8.8 (6.2,12.3)	9.2 (6.4,12.2)	8.9 (6.3,12.6)
HB (g/dL)	10.1 (8.7,11.9)	10.1 (9.0,11.6)	10.2 (9.2,11.3)	10.2 (9.1,11.3)	11.2 (10.2,12.7)	11.3 (10.2,12.5)
MCH (pg)	30.5 (29.0,31.5)	30.2 (28.9,31.6)	30.4 (29.1,31.5)	30.2 (29.0,31.6)	30.7 (29.4,31.9)	30.4 (29.3,31.8)
MCHC (%)	33.2 (32.2,34.0)	33.2 (32.4,34.0)	33.2 (32.4,34.1)	33.2 (32.3,34.0)	33.6 (32.8,34.5)	33.6 (32.7,34.3)
INR (s)	1.3 (1.2,1.5)	1.4 (1.2,1.5)	1.3 (1.2,1.4)	1.3 (1.2,1.4)	1.4 (1.3,1.6)	1.4 (1.3,1.6)
PT (s)	14.7 (12.9,16.5)	14.8 (12.9,16.3)	14.2 (13.2,15.6)	14.3 (13.1,15.5)	15.6 (14.3,17.3)	15.7 (14.2,17.2)
APTT (s)	37.6 (29.4,58.6)	42.1 (29.7,61.0)	33.5 (29.3,41.2)	33.7 (29.3,42.8)	40.7 (31.4,62.0)	45.1 (31.5,64.0)
BUN (mg/dL)	16.0 (13.0,22.0)	16.0 (13.0,22.0)	16.0 (12.5,20.5)	16.0 (13.0,21.0)	16.0 (13.0,22.0)	16.0 (13.0,22.0)
Creatinine (mg/dL)	0.9 (0.7,1.1)	0.9 (0.8,1.1)	0.8 (0.7,1.0)	0.9 (0.7,1.1)	0.9 (0.7,1.1)	0.9 (0.8,1.2)
Serum Potassium (mg/dL)	4.2 (3.9,4.5)	4.2 (4.0,4.5)	4.2 (4.0,4.5)	4.2 (4.0,4.5)	4.4 (4.1,4.70	4.5 (4.2,4.7)
PO2 (mmHg)	373.0 (306.2,424.0)	368.0 (301.0,419.0)	247.7 (209.7,285.5)	240.8 (209.2,281.9)	405.0 (358.2,447.0)	401.0 (359.2,442.7)
PCO2 (mmHg)	40.0 (37.0,44.0)	41.0 (37.0,44.0)	40.4 (38.0,43.0)	40.5 (38.2,42.8)	47.0 (43.0,51.0)	47.0 (44.0,51.0)
PaO2FiO2Ratio	268.0 (179.2,372.2)	259.5 (177.2,373.3)	267.1 (205.1,339.4)	252.0 (196.1,331.5)	326.0 (254.0,417.5)	321.8 (248.0,410.0)
Lactate (mmol/L)	1.3 (1.0,1.7)	1.3 (1.0,1.7)	1.8 (1.4,2.3)	1.8 (1.5,2.2)	2.4 (1.8,3.1)	2.4 (1.9,3.2)
Events						
AKI (%)	78.5	79.6	78.5	79.6	78.5	79.6
Length of ICU (days)	2.0 (1.2,3.1)	2.0 (1.2,3.2)	2.0 (1.2,3.2)	2.0 (1.2,3.2)	2.0 (1.2,3.1)	2.0 (1.2,3.2)
Hospital mortality (%)	0.8	1.0	0.8	1.0	0.8	1.0
AKI stage (%)						
AKI stage 1	26.1	27.3	26.1	27.3	26.1	27.3
AKI stage 2	45.0	44.1	45.0	44.1	45	44.1
AKI stage 3	7.4	8.2	7.4	8.2	7.4	8.2

BMI, body mass index; SOFA, Sequential Organ Failure Assessment; GCS, Glasgow Coma Scale; CKD, CKD, Chronic Kidney Disease; WBC, white blood cell count; RBC, red blood cell count; HB, hemoglobin; MCH, mean erythrocyte hemoglobin content; MCHC, mean erythrocyte hemoglobin concentration, platelet count; INR, international normalized ratio; PT, plasminogen time; ALP, alkaline phosphatase; BUN, blood urea nitrogen; MO, monocyte count; NEUT, neutrophil count; APTT, activated partial thromboplastin time; PO2, partial pressure of oxygen; PCO2, partial pressure of carbon dioxide; PaO2FiO2Ratio, oxygenation index; AKI, acute kidney injury; ICU, intensive care unit.

Table 1 shows that 719 (78.5%) patients met the KDIGO criteria for AKI in the training cohort, while 309 (79.6%) patients met the criteria for AKI in the validation cohort. The median ages of these patients were 69.8 years (IQR: 61.9, 77.1) and 69.7 years (IQR: 61.4, 77.4), respectively. The in-hospital mortality rates for all included patients were 0.8% (7/916) and 1.0% (4/388), and the numbers of days in the ICU were 2.0 days (IQR: 1.2, 3.1) and 2.0 days (IQR: 1.2, 3.2), respectively.

### Construction of models for predicting the risk of AKI occurrence

First, univariate analysis was carried out for laboratory indicators with less than 20% missing values to screen for significant independent risk factors (p < 0.05) that could serve as potential predictor variables. Then, we performed the following three regression analyses on the screened potential predictor variables to construct prediction models.

1 Multivariate logistic-LR regression analysis

Multivariate logistic regression analysis was performed on the potential predictors screened for each of the three datasets based on univariate analysis. The results showed that the potential predictor variables for Q1 were age, weight, systolic blood pressure, mean arterial pressure, diabetes mellitus, atrial fibrillation, preoperative hemoglobin, preoperative creatinine, and postoperative neutrophil counts; the potential predictor variables for Q2 were age, weight, mean arterial pressure, atrial fibrillation, diabetes mellitus, preoperative hemoglobin, and postoperative neutrophil counts; the potential predictor variables for Q3 were age, weight, mean arterial pressure, atrial fibrillation, preoperative hemoglobin, preoperative creatinine, preoperative glucose, postoperative neutrophil counts, and postoperative international normalized ratio, with atrial fibrillation and diabetes mellitus as categorical variables. Moreover, among arterial pressure, atrial fibrillation, preoperative hemoglobin, preoperative creatinine, preoperative glucose, postoperative neutrophil count, and postoperative International Normalized Ratio, atrial fibrillation and diabetes mellitus were categorical variables, and the remaining variables were continuous (Table 1). The VIFs of the latent variables ultimately included in the three models were all less than 2, which shows that the covariances among the variables were all weak (S2 Table).

2 LASSO regression analysis

**Fig 2:**
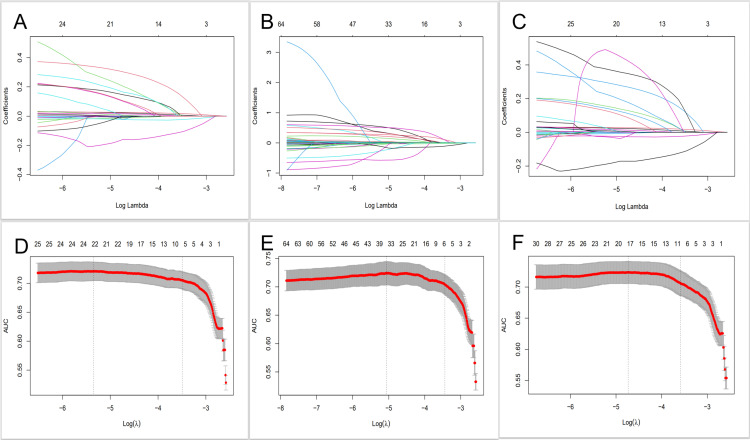
A, Coefficient profile plot for Q1; B, Coefficient profile plot for Q2; C, Coefficient profile plot for Q3; D, Lasso regression variable trajectories for Q1; E, Lasso regression variable trajectories for Q2; F, Lasso regression variable trajectories for Q3. Predictor selection by the least absolute shrinkage and selection operator (LASSO) regression method.

To construct a LASSO regression model for predicting CSA-AKI, all potential predictors were included in the LASSO regression analysis. We used the minimum standard and minimum standard 1x standard error of ten cross-validations in LASSO regression analysis to select the optimal lambda to minimize model bias. As shown in Fig 2, a total of 6 independent risk factors were screened in Q1, namely, age, weight, mean arterial pressure, atrial fibrillation, preoperative hemoglobin and postoperative partial pressure of oxygen; a total of 6 independent risk factors were screened in Q2, namely, age, weight, mean arterial pressure, atrial fibrillation, preoperative hemoglobin and postoperative monocyte count; and a total of 10 independent risk factors were screened in Q3, namely, age, weight, mean arterial pressure, diabetes mellitus, atrial fibrillation, preoperative hemoglobin, preoperative creatinine, preoperative glucose, postoperative monocyte count and postoperative international normalized ratio. Similarly, we analyzed the covariance of the variables included in the Lasso model, and the results showed that the variance inflation factor (VIF) values for each variable were less than 5 (S3 Table).

3 Random forest (RF)

RF is one of the most advanced multiple machine learning (ML) methods, and Junlong Hu et al.‘s study concluded that the RF model has the strongest discriminative ability for AKI in critically ill children [11]. Random forests are nonparametric methods that can be adapted to different types of responses, such as categorical or quantitative outcomes and survival times; moreover, these methods are well suited for analyzing complex data and can highlight the relevance of each predictor through so-called variable importance measures [12]. In this study, we used all significantly different latent variables for random forest analysis and constructed optimal random forest models for Q1, Q2, and Q3 based on the correlation of predictors, as shown in S5 Table. The VIFs for each variable included in the three RF models were less than 5 (S4 Table).

We summarized the results of the above regression analyses and ultimately created a total of nine CSA-AKI candidate predictive models based on the three data sets. In addition we included the SOFA score [13], a predictor variable in the Rapid Acute Kidney Injury Score, to predict the incidence of AKI in patients admitted to the ICU after cardiac surgery (S5 Table).

### Evaluating the validity of the predictive models

**Fig 3:**
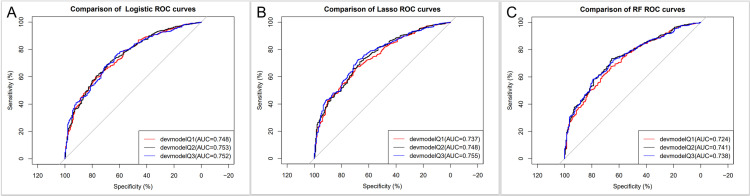
A, ROC curves for Logistic models constructed on the basis of three cohort groups; B, ROC curves for Lasso models constructed on the basis of three cohort groups; C, ROC curves for RF models constructed on the basis of three cohort groups. RF, random forest.

In this study, a total of 9 prediction models were constructed, and the ROC curves of the prediction models were plotted. The area under the ROC curve (AUROC) is defined as the area under the ROC curve. In general, a larger AUROC indicates a greater accuracy of the prediction model and a better diagnostic performance. As shown in Table 2, the AUCs of the nine models were greater than 0.7, and the AUCs (95% CIs) of the three models (logistic, LASSO, and RF) in Q1 were 0.748 (0.7109 ~ 0.7853), 0.737 (0.6998 ~ 0.7747), and 0.724 (0.6863 ~ 0.7626), respectively; the AUCs (95% CIs) of the three models in Q2 (logistic, LASSO, and RF) were 0.753 (0.7156 ~ 0.7897), 0.748 (0.7105 ~ 0.7847), and 0.741 (0.7044 ~ 0.7781), respectively; the AUC (95% CI) of the 3 models (logistic, LASSO, and RF) in Q3 were 0.753 (0.7156 ~ 0.7897), 0.748 (0.7105 ~ 0.7847), and 0.741 (0.7044 ~ 0.7781), respectively; and the AUC (95% CI) were 0.752 (0.7152 ~ 0.7883), 0.755 (0.7185 ~ 0.7912), and 0.738 (0.7018 ~ 0.7752). We plotted the corresponding ROC curves (Fig 3). Fig 3A depicts the comparison of ROC curves for prediction models derived from the Logistic regression algorithm across three distinct groups (Q1, Q2, Q3). Here, the abscissa denotes specificity, while the ordinate represents sensitivity. A curve positioned closer to the upper-left corner signifies stronger discriminatory capacity for the target outcome. As illustrated in the legend, the AUC values for devmodelQ1, devmodelQ2, and devmodelQ3 were 0.748, 0.753, and 0.752, respectively. Notably, the Logistic models in Q2 and Q3 groups exhibited significantly higher AUCs compared to Q1, indicating superior diagnostic efficacy in these subgroups. This suggests that the Logistic model more accurately discriminates between positive and negative outcomes in Q2 and Q3 scenarios, offering a robust foundation for clinical decision-making. Fig 3B illustrates the ROC curve analysis for the Random Forest (RF) model across Q1, Q2, and Q3. The AUCs for devmodelQ1, devmodelQ2, and devmodelQ3 were 0.724, 0.741, and 0.738, respectively. While all three groups demonstrated AUC values exceeding 0.7-indicating baseline diagnostic utility-the RF model in Q1 yielded the lowest AUC, whereas the Q2 group’s model showed comparatively superior performance. Clinically, these results emphasize the importance of selecting RF models tailored to specific scenarios; the Q2 model’s balanced sensitivity and specificity profile may optimize predictive accuracy in corresponding clinical contexts. Fig 3C displays the ROC curves for the LASSO model across the three groups. The AUCs for devmodelQ1, devmodelQ2, and devmodelQ3 were 0.737, 0.748, and 0.755-the latter being the highest among all models analyzed. The Q3 group’s LASSO model, with an AUC of 0.755, demonstrated significantly improved discrimination of diseased versus non-diseased populations compared to Q1. From a clinical research standpoint, this model’s exceptional performance positions it as a top candidate for supporting clinical decisions, particularly in scenarios analogous to the Q3 subgroup. We screened out the first three models according to the AUC value of two of the logistic model, it can be seen that compared with the newer machine learning model, in certain scenarios, the performance of the model established by traditional statistical analysis methods may be more, so when we build a predictive model to select statistical analysis methods, machine learning methods can not yet completely replace the traditional statistical analysis methods.

**Table 2:** Predictive performance of CSA-AKI’s nine predictive modeling systems in a three -group cohort.

**Q1**					
**MODEL**	AUC	95%CI	Specificity	Sensitivity	Youden’s index
**LOGISTIC**	0.748	0.7109-0.7854	0.7766497	0.596662	0.3733118
**LASSO-LOGISTIC**	0.737	0.6998-0.7747	0.6903553	0.6675939	0.3579492
**RADOM FOREST**	0.724	0.6863-0.7626	0.6598985	0.6759388	0.3358373
**Q2**					
**MODEL**	AUC	95%CI	Specificity	Sensitivity	Youden’s index
**LOGISTIC**	0.753	0.7156-0.7897	0.6903553	0.689847	0.3802023
**LASSO-LOGISTIC**	0.748	0.7105-0.7847	0.6649746	0.7093185	0.3742931
**RADOM FOREST**	0.741	0.7044-0.7781	0.6548223	0.7343533	0.3891756
**Q3**					
**MODEL**	AUROC	95%CI	Specificity	Sensitivity	Youden’s index
**LOGISTIC**	0.752	0.7152-0.7883	0.6192893	0.7635605	0.3828498
**LASSO-LOGISTIC**	0.755	0.7185-0.7912	0.680203	0.7232267	0.4034297
**RADOM FOREST**	0.738	0.7018-0.7752	0.7918782	0.5827538	0.374632

AUROC, the area under the receiver operating characteristic curves; CI, confidence interval. The models’ performance was analyzed by the Hosmer-Lemeshow (H-L) test and ROC analysis.

**Fig 4:**
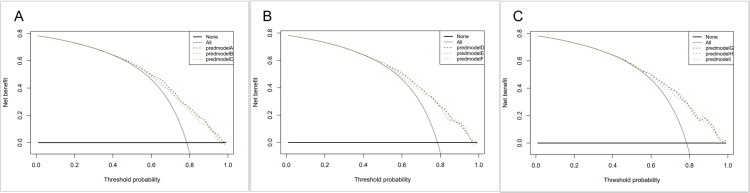
A, The results of the decision curve analysis for group Q1; B, The results of the decision curve analysis for group Q2; C, The results of the decision curve analysis for group Q3. PredmodelA, Logistic model of Q1; predmodelB, LASSO model of Q1; predmodelC, RF model of Q1; predmodelD, Logistic model of Q2; predmodelE, LASSO model of Q2; predmodelF, RF model of Q2; predmodelG, Logistic model of Q3; predmodelH, LASSO model of Q3; predmodelI, RF model of Q3.

Decision curve analysis (DCA) is a simple method for evaluating clinical predictive models, diagnostic tests, and molecular markers that integrates and analyses patient or decision-maker preferences while meeting the practical needs of clinical decision-making. As shown in the figure, in the DCA curve, the grey diagonal curve indicates intervention for all patients, the grey parallel line indicates no intervention for any patient, and the red, black, and green colors indicate the clinical benefit of the logistic, LASSO, and RF models, respectively (Fig 4). Combining the DCA curves of the nine prediction models (Fig 4), we can see that postoperative AKI risk assessment using the prediction models in this study yields some net benefit within most prediction thresholds.

High specificity means is well known to indicate that the model has high accuracy in identifying actual negative samples. High sensitivity means that the model has high accuracy in identifying actual positive samples. A model with high sensitivity in clinical applications can minimize missed diagnoses. Youden’s index is a statistical metric used to assess the performance of a diagnostic test, with a larger Youden’s index indicating better performance and greater fidelity of the classification model. The specificity, sensitivity, and Youden’s index of the nine predictive models constructed from the three sets of data are shown in Table 2. A comparison of the three sets of data revealed that the RF model of Q3 had the highest specificity (0.7918782), followed by the logistic model of Q1 (0.7766497); the logistic model of Q3 had the highest sensitivity (0.7635605), followed by the RF model of Q2 (0.7343533). The LASSO model of Q3 had the highest Youden index of 0.4034297.

### Validation of predictive models

The top three models ranked according to the AUC value were the LASSO model based on Q3, the logistic model based on Q2, and the logistic model based on Q3, while the model with the lowest AUC value was the RF model based on Q1. The main principle of the NRI and IDI is based on the “gold standard”, which is to reclassify the classification results of a model according to a new set of criteria and to examine the ability of the reclassified metrics to improve. According to further analysis of the IDI values, the LASSO model of Q3 improved the predictive ability by 3.12% (95% CI: 1.78% to 4.46%) compared to that of the Q1 random forest model; the logistic model of Q2 improved the predictive ability by 3.49% (95% CI: 1.88% to 4.49%) compared to that of the Q1 random forest model; and the Q3 logistic model improved the predictive ability by 2.88% (95%:1.6% to 4.16%) over that of the Q1 random forest model.

In the calibration curve, the vertical coordinate represents the probability of actual occurrence in the study cohort, and the vertical coordinate represents the estimated probability of the model; a well-calibrated model should try to keep the calibration curve as close as possible to the ideal line, which indicates that the predicted probability of the model matches the probability of actual occurrence.

**Fig 5:**
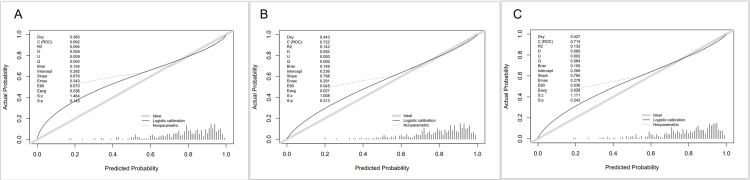
A, Calibration curves for Q2’s logistic model; B, Calibration curves for Q3’s logistic model; C, Calibration curves for Q3’s LASSO model. Calibration plots illustrate the relationship between the predicted AKI risk according to the models and the actual occurrence of AKI in the validation data. Plots along the 45 line indicate model calibration, the more similar the predicted probability is to the actual outcome, the better the dashed line fits the solid line, indicating a better predictive model.

We plotted the calibration curves for the LASSO model of Q3, the logistic model of Q2, and the logistic model of Q3 based on the validation set of data, as shown in Fig 5. Panels A, B, and C respectively display the calibration curves of different models. The vertical axis represents the actual occurrence probability (Actual Probability), while the horizontal axis indicates the model-predicted probability (Predicted Probability). The figure contains three curves: the gray “Ideal line (Ideal)” represents the ideal state where the predicted probability aligns perfectly with the actual probability; the black “Logistic calibration” denotes the logistic calibration curve, reflecting the calibration status of the logistic regression model; the dashed “Nonparametric” serves as the non-parametric calibration curve, illustrating the relationship between predictions and actual outcomes from a non-parametric perspective. Statistics such as Dxy, C (ROC), and R^2^ listed in the figure are utilized to quantify the models’ performance. As shown in the figure, compared with the logistic model of Q2, the probabilities estimated by the LASSO model and the logistic model built based on Q3 have greater conformity and better consistency with the actual values. Although the sensitivity and Youden’s index of Q3’s logistic model are slightly lower than those of Q3’s LASSO model, the calibration curves of Q3’s logistic model are closer to the ideal curves, and Q3’s logistic model is simpler and easier to use clinically.

Summarizing the above consistency test indexes, the logistic model based on the data of Q3 group not only has a high AUC value (0.752) among the nine prediction models, but also ranks the top three in sensitivity and Jordon’s index among the nine prediction models. Combined with Fig 4, the logistic model based on the Q3 group data is not only simpler and easier to operate than the other prediction models, but also has greater conformity and better consistency with the actual values. And as shown in Fig 5, it also has a certain clinical net gain in actual clinical use. Therefore, we finally selected the prediction model as the logistic model based on the data of Q3 group.

### Nomogram establishment

**Fig 6:**
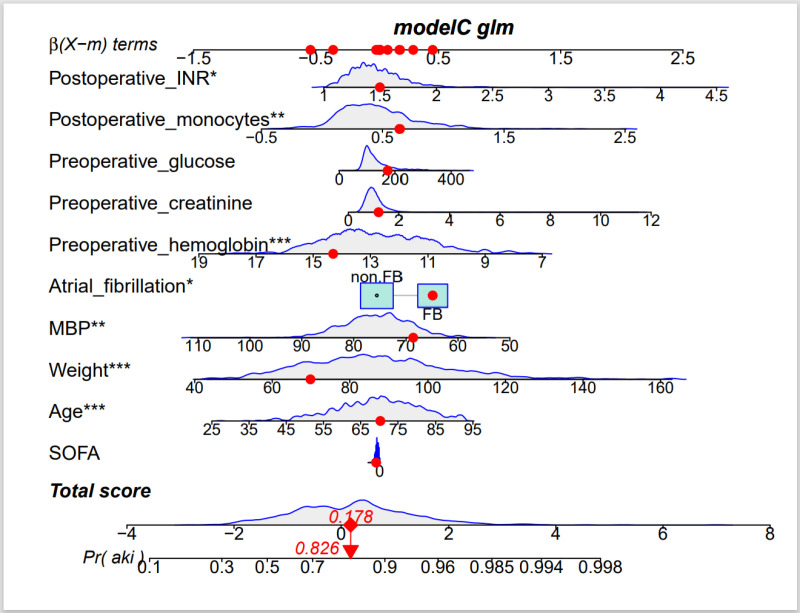
Nomogram to predict the incidence of postoperative AKI in patients undergoing cardiac surgery. AKI, Acute Kidney Injury; MBP, mean arterial pressure; SOFA, Sequential Organ Failure Assessment.

We plotted nomogram column-line plots based on the regression coefficients for each variable of the logistic prediction model for Q3, as shown in Fig 6. The scores of each variable were obtained from the specific values of each variable and summed to obtain the total score, which was converted to the probability of occurrence of CSA-AKI in the patient by the probability axis of the nomogram. Based on this model, we constructed a nomogram to predict the risk of CSA-AKI. To illustrate its application, we randomly selected a patient and plotted their clinical parameters on the nomogram to compute the total score and corresponding CSA-AKI risk. The patient’s preoperative data included a glucose level of 174, creatinine of 1.2, and hemoglobin of 14.3 within 24 hours before surgery, along with baseline characteristics: a history of atrial fibrillation, mean arterial pressure (MBP) of 68.64, weight of 69.85, age of 70.28, and a SOF score of 5. Postoperatively, their INR was 1.5 within 48 hours, and monocyte count was 0.64. Summing these factor scores yielded a total of 0.178, translating to an approximately 82.6% risk of developing CSA-AKI.

## Discussion

Although the pathophysiological mechanism of postoperative AKI after cardiac surgery has not been clarified thus far, many clinical data and bioindicators have been proven to have some early predictive value for CSA-AKI. These methods have been applied to construct prediction models by other authors.

These factors have been reported as potential predictors, including sex, age, height, weight, BMI, body temperature, heart rate, respiratory rate, systolic blood pressure, diastolic blood pressure, oxygen saturation, obesity, renal disease without RRT, hypovolemia, hepatorenal syndrome, diabetes mellitus, cerebrovascular disease, malignancy, prior chronic kidney disease, sepsis, shock, multiorgan failure, Charlson comorbidity index (≥2 points), LVEF ≤30%, NYHA score (>2 points), previous cardiac surgery [14], valve and coronary artery bypass grafting, cardiopulmonary bypass application, cardiopulmonary bypass duration, mechanical ventilation [15], dialysis, red blood cell transfusion [16], HDL cholesterol concentration [17], proteinuria [18], preoperative glomerular filtration rate [19], urinary liver-type fatty acid binding protein (L-FABP), urinary neutrophil gelatinase-associated lipocalin, serum L-FABP, urinary interleukin-18, urinary kidney injury molecule-1 [20], neutrophil-lymphocyte ratio [21], white blood cell count, hemoglobin, erythrocyte pressure volume, platelet count, blood creatinine, blood urea nitrogen, bicarbonate, and serum potassium [22].

Based on the prediction model assessment time, we can broadly categorize several identified models for AKI risk assessment after cardiac surgery into preoperative, intraoperative, and postoperative models. And the pathogenesis of AKI can occur in preoperative, intraoperative as well as postoperative still and phases or simultaneously in all three phases at the same time [16,23–25]. In our study, a prediction model for AKI associated with cardiac surgery was developed and validated using routine laboratory test indices 48 hours before and up to 24 hours after surgery, as well as clinical data and scores of patients. The aim of this study is to provide an early risk assessment of patients undergoing cardiac surgery without additional invasive procedures and without increasing the risk of surgery or the cost of hospitalization, using available preoperative and postoperative laboratory indicators, clinical scores, and patient baseline data, in order to achieve timely intervention, shorten the length of hospital stay, and reduce the total cost of hospitalization. However, patients undergoing cardiac surgery usually have more than one routine laboratory index in the 48 hours prior to surgery, as well as surgical patients admitted to the ICU who may also have multiple routine laboratory indexes reviewed, usually in the 24 hours after surgery, due to the need to assess changes in the patient’s condition. Our search of the MIMIC IV database revealed that each patient enrolled usually had more than one record of routine laboratory tests that met the screening time criteria (48 hours preoperatively and up to 24 hours postoperatively). A review of the relevant literature found that the first preoperative, intraoperative, and postoperative laboratory indicators were generally used to establish CSA-AKI prediction models [7,26]. There is also related literature on the use of the MIMIC database to build predictive models by selecting the average value of the experimental indicators [8]. Compared with the published literature, we have added the grouping of the maximum values of laboratory indicators in the 48 hours before and 24 hours after the operation, and we have used three statistical methods to establish a prediction model for comparative analysis, which effectively avoids the chance of analyzing the results and improves the accuracy and credibility of the results.

In this study, we visually demonstrate the diagnostic performance of logistic regression, random forest, and LASSO models across different cohorts through ROC curves. As a gold-standard metric in ROC analysis, the area under the curve (AUC) quantifies model accuracy by summarizing the trade-off between sensitivity and specificity. The findings validate several core principles in medical ROC analysis: 1) higher AUC values—such as those observed in the Q3 LASSO model and Q2/Q3 logistic regression models—correspond to superior diagnostic utility; 2) model performance can vary significantly across subgroups, necessitating tailored model selection; and 3) traditional statistical methods (e.g., logistic regression) can rival modern machine learning approaches in specific clinical scenarios. These insights not only provide guidance for researchers to select the most appropriate predictive models but also highlight the importance of integrating ROC results with the clinical and biological characteristics of the studied populations.

As depicted in Fig 5, Panel A illustrates the calibration curve of the Q2 logistic model. With an ROC value of 0.692, this model exhibits moderate discriminatory capacity for target events (e.g., AKI). Panel B showcases the Q3 logistic model, characterized by an ROC value of 0.722 and a Dxy value of 0.443—metrics indicative of enhanced discrimination between positive and negative samples. Panel C presents the Q3 LASSO model, with an Eavg of 0.026, suggesting a relatively minimal overall prediction bias. Upon comparative analysis of the calibration curves across these three predictive models, the calibration curve of the Q3 logistic model demonstrates closer alignment with the ideal line. This observation signifies a higher degree of consistency between the predicted probability and the actual occurrence probability. Consequently, the Q3 logistic model not only exhibits superior clinical prediction reliability but also provides a more robust foundation for risk assessment, enabling effective clinical risk stratification. Such findings underscore the Q3 logistic model’s utility in translating predictive analytics into actionable clinical decisions for target events like AKI.

Multivariate logistic regression (LR) models are well-suited for scenarios where data exhibit a clear linear relationship and there is a high demand for model interpretability. For instance, in medical research, when predicting the relationship between the occurrence of a disease and known risk factors, logistic regression can clearly explain the impact of each factor on the probability of disease occurrence [27,28]. Lasso regression is appropriate for high-dimensional data with feature redundancy, particularly when aiming to perform feature selection to enhance model generalization. For example, in gene expression data analysis, Lasso regression can identify key genes associated with a disease by shrinking the coefficients of irrelevant genes, thereby achieving feature selection [29]. Random forest (RF) models generally perform better in handling complex nonlinear problems, especially when dealing with large datasets and prioritizing model accuracy. In fields such as image recognition and speech recognition, random forests can manage highly complex feature relationships and achieve high recognition accuracy [26,30]. Machine learning modelling methods such as RF can rank the importance of the variables in the model. Furthermore, Po-Yu Tseng’s study showed that the models constructed by machine learning outperform the models constructed by traditional regression analysis methods in predicting CSA-AKI with small sample sizes. However, comparing the models constructed in this study using three different methods showed that logistic regression, a traditional regression analysis method, outperforms the models constructed by traditional regression analysis methods. Traditional logistic regression analysis methods are superior to machine learning methods such as RF. In Penghua Hu’s study, the final selection of predictive models was also constructed by multifactor logistic regression analysis [31]. Therefore, we believe that the traditional logistic regression model remains superior for cases involving many potential variables and samples.

The final model developed included patient age, weight, mean arterial pressure, atrial fibrillation, preoperative hemoglobin, preoperative creatinine, preoperative glucose, postoperative mononuclear cell count, postoperative INR, and the SOFA score. The nomogram also demonstrated a high degree of predictive ability in the validation cohort. This model is simple and easy to operate and has some net clinical benefit with a high AUC value (0.752), sensitivity (0.7635605), and Jordon’s index (0.3828498) is simpler and easier to operate and has good conformity and consistency with the actual values. Clinical implementation of the nomogram involves systematically inputting validated preoperative and postoperative variables into its computational framework. Specifically, this includes integrating baseline demographics (age, weight, atrial fibrillation history), hemodynamic parameters (mean arterial pressure), preoperative laboratory maxima (hemoglobin, creatinine, glucose), and postoperative laboratory maxima (monocyte count, INR). The nomogram generates a risk score that provides real-time, point-of-care decision support. And this score allows clinicians to dynamically stratify patients into distinct risk categories and implement immediate, risk-tailored actions, such as intensifying renal monitoring, initiating preemptive nephroprotective measures, or consulting nephrology early. The model represents a paradigm shift in clinical practice by offering a quantitative, evidence-based risk assessment that complements traditional subjective evaluations. By objectively categorizing patients into high- and low-risk groups for CSA-AKI, the nomogram enables data-driven, proactive decisions. This approach reduces reliance on anecdotal reasoning, enhances risk stratification accuracy, and ultimately facilitates timely, personalized interventions to improve patient outcomes.

Comparing the nine risk prediction models we developed shows that preoperative hemoglobin is a significant predictor in patients with CSA-AKI. Kulier A et al. suggested that a low preoperative hemoglobin level is an independent predictor of poor noncardiac prognosis, including AKI [32]. A study that included 123 consecutive patients who underwent pulmonary endarterectomy (PEA) also demonstrated that a low preoperative hemoglobin concentration was an independent risk factor for AKI after PEA. Congya Zhang and Guyan Wang deduced the underlying mechanism in their article. They concluded that the hemoglobin concentration determines arterial blood oxygen content, which plays an important role in tissue oxygen supply. Therefore, a lower hemoglobin concentration reduces oxygen delivery to the kidneys, predisposing them to renal medullary injury and ultimately leading to exacerbation of postoperative AKI [33]. In another study that included 920 patients who underwent cardiac surgery combined with extracorporeal circulation, the authors concluded that the incidence of AKI significantly increased when hemoglobin levels were extremely low (<25%) [34].

Our study also has shortcomings. First, this study only used the creatinine item of KDIGO as a diagnostic criterion for postoperative AKI and did not include urine output as a diagnostic criterion; therefore, it may have underestimated the incidence of postoperative AKI. Second, this study only used the MIMIC-IV database, which is based on the U.S. population; therefore, the applicability of the prediction model to other populations remains to be validated. Third, due to the limitations of the database itself, some of the latent variables with missing records could not be included in the statistical analysis.

## Conclusion

In this study, we developed nine early predictive models for CSA-AKI using logistic regression, LASSO regression, and random forest regression. Comparative analysis revealed that traditional logistic regression retained notable advantages over machine learning methods like random forest, indicating modern techniques cannot fully replace traditional regression. Models based on maximum laboratory values outperformed those using other datasets. Integrating statistical and clinical insights, we identified the logistic regression model incorporating preoperative and postoperative maxima maxima as optimal. This model includes: 1) baseline characteristics (age, weight, mean arterial pressure, SOFA score, atrial fibrillation history); 2) preoperative 48-hour maxima of hemoglobin, creatinine, and glucose; and 3) postoperative 24-hour maxima of monocytes and INR. Its key strength lies in synthesizing perioperative dynamic variables, capturing both baseline risks and acute intraoperative/postoperative physiological changes. This framework offers a robust tool for early CSA-AKI risk assessment.

## Supporting information

S1 Table Variability between modelling and validation groups. (PNG)

S2 Table VIF values for logistic prediction models. (PNG)

S3 Table VIF values for lasso prediction models. (PNG)

S4 Table VIF values for random forest prediction models. (PNG)

S5 Table Predictors included in the models. (PNG)

S1 Data Raw data. (ZIP)
