# Supplementary figures and images for: Development and validation of a cardiac surgery-associated acute kidney injury prediction model using the MIMIC-IV database
Source: PLoS One. 2025 Jun 12;20(6):e0325151. doi: 10.1371/journal.pone.0325151 (PMC12161578; doi:10.1371/journal.pone.0325151)

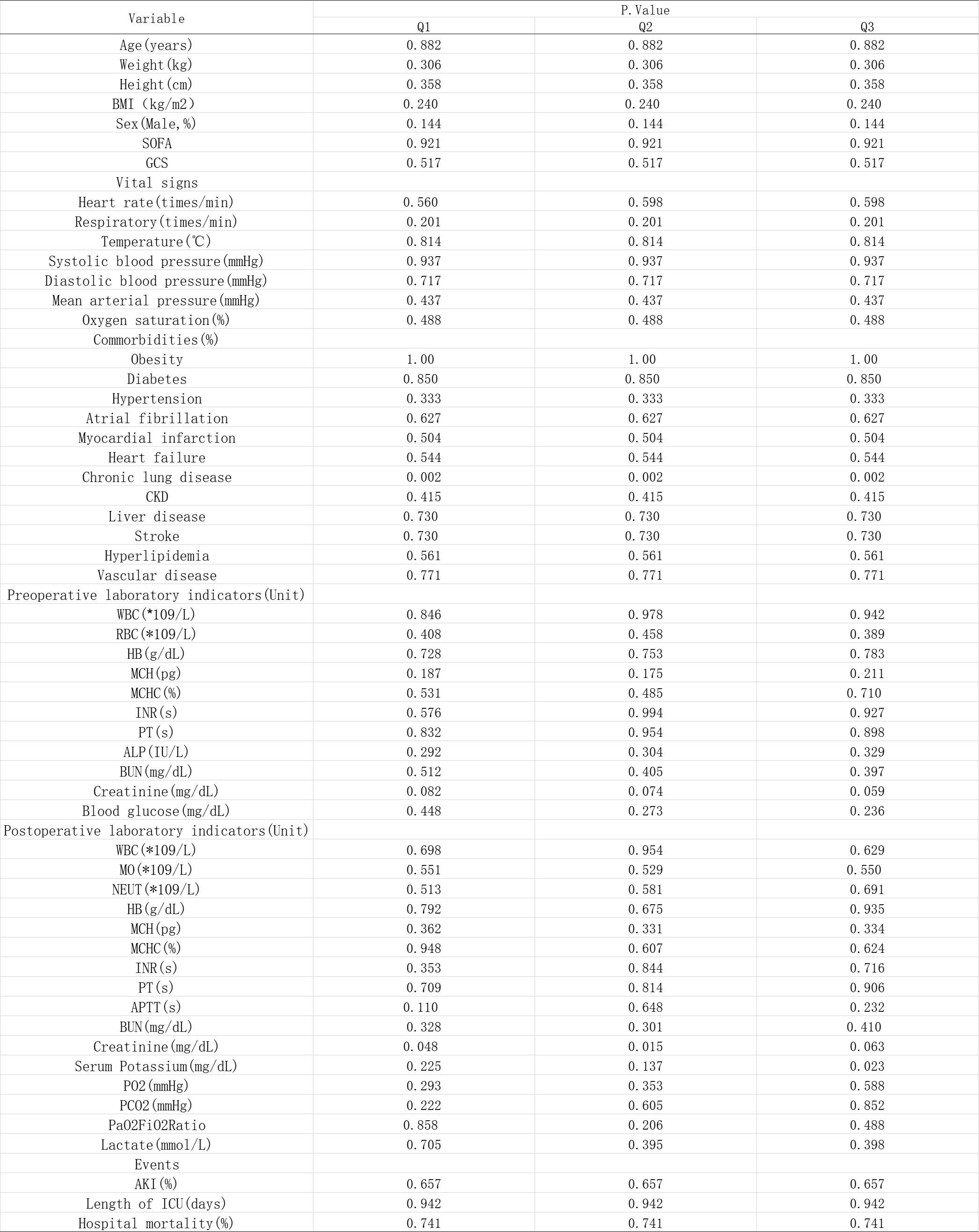

Supplement: S1 Table — (PNG) [file pone.0325151.s001.png]

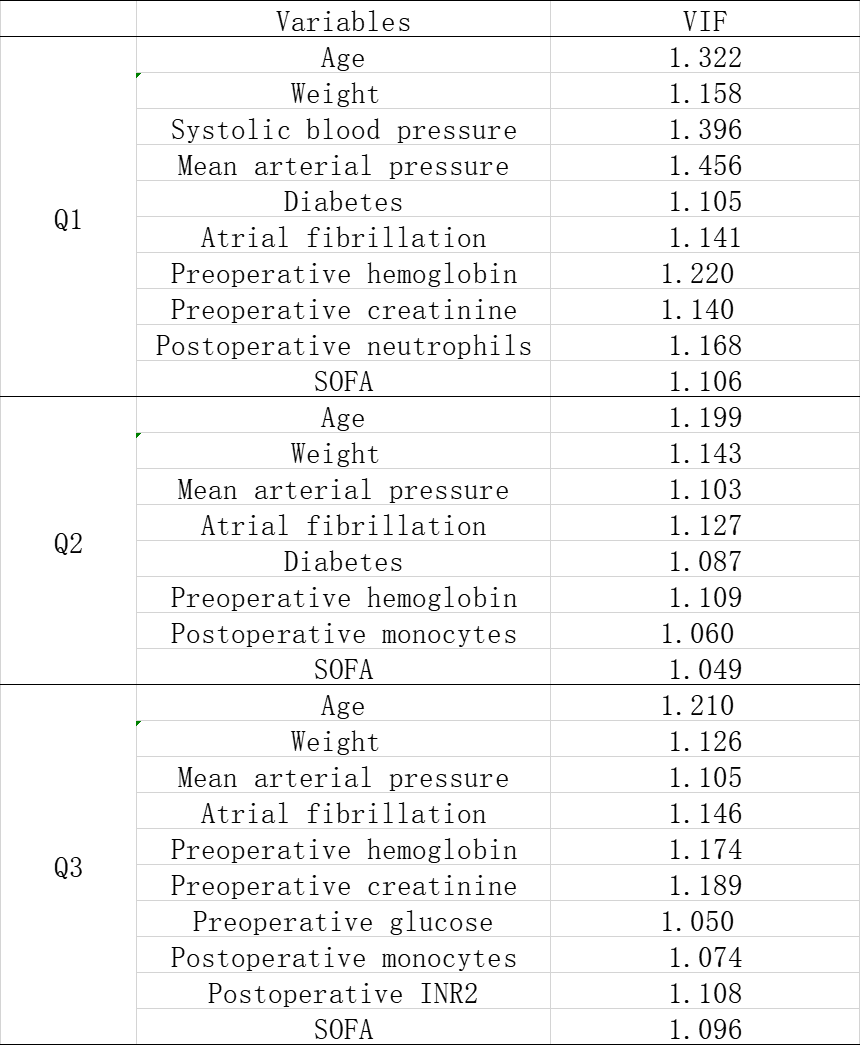

Supplement: S2 Table — (PNG) [file pone.0325151.s002.png]

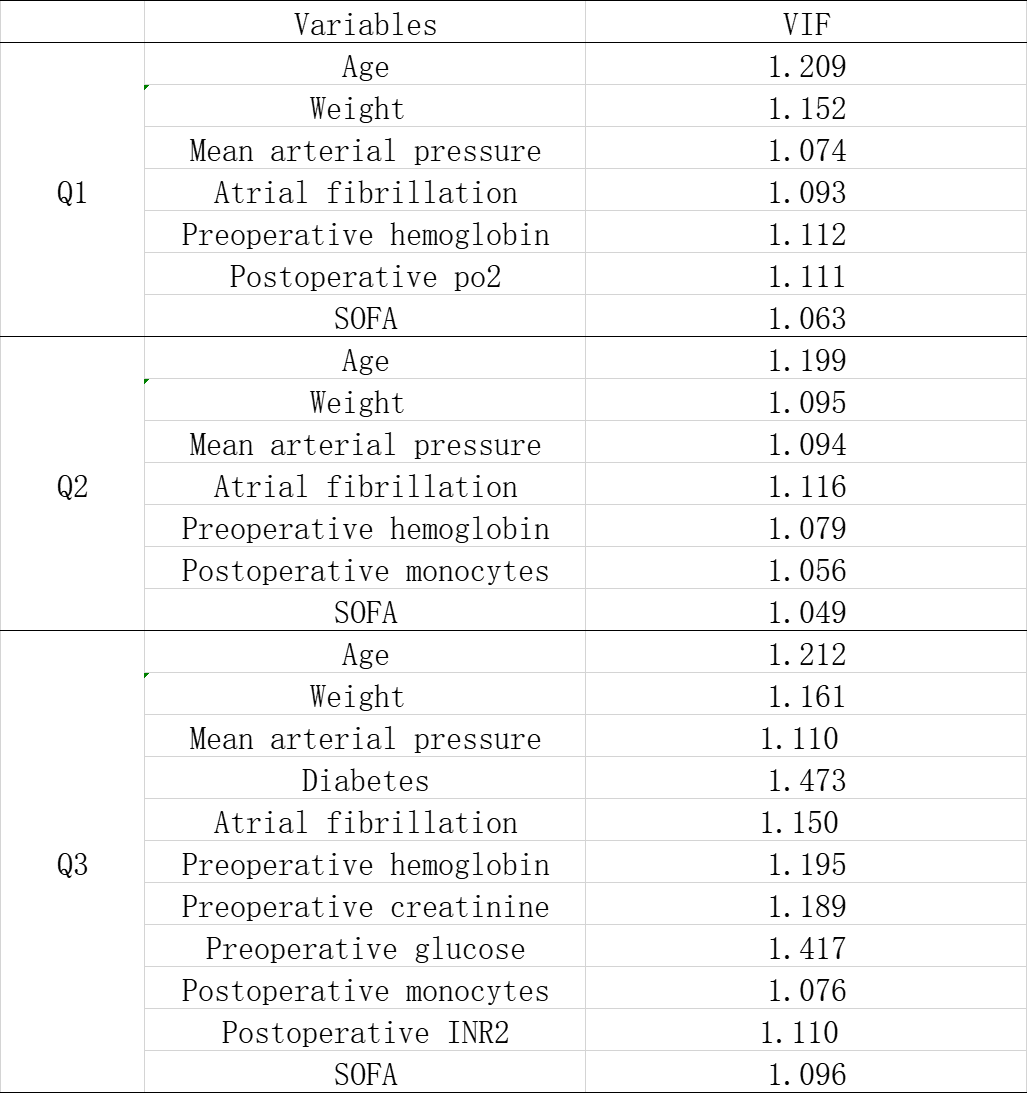

Supplement: S3 Table — (PNG) [file pone.0325151.s003.png]

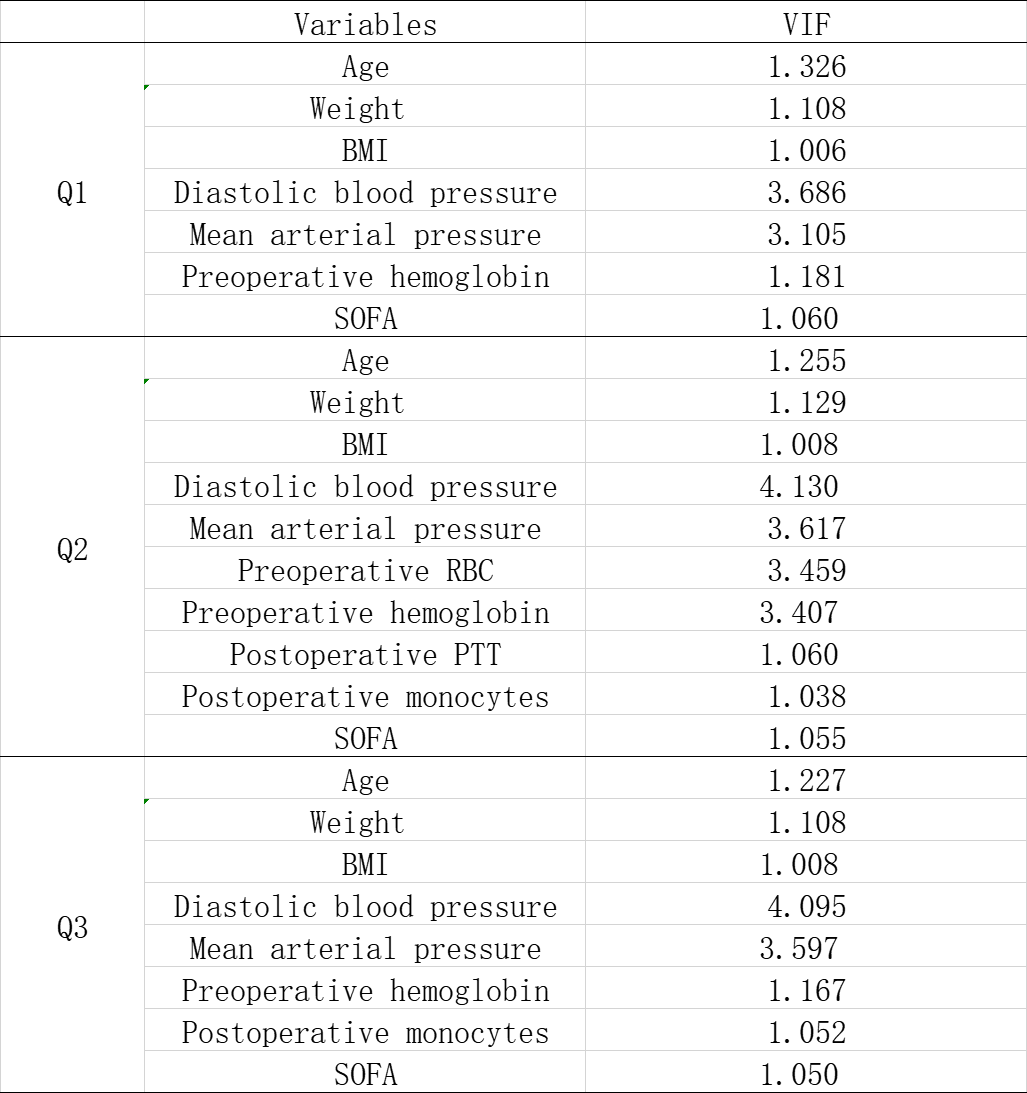

Supplement: S4 Table — (PNG) [file pone.0325151.s004.png]

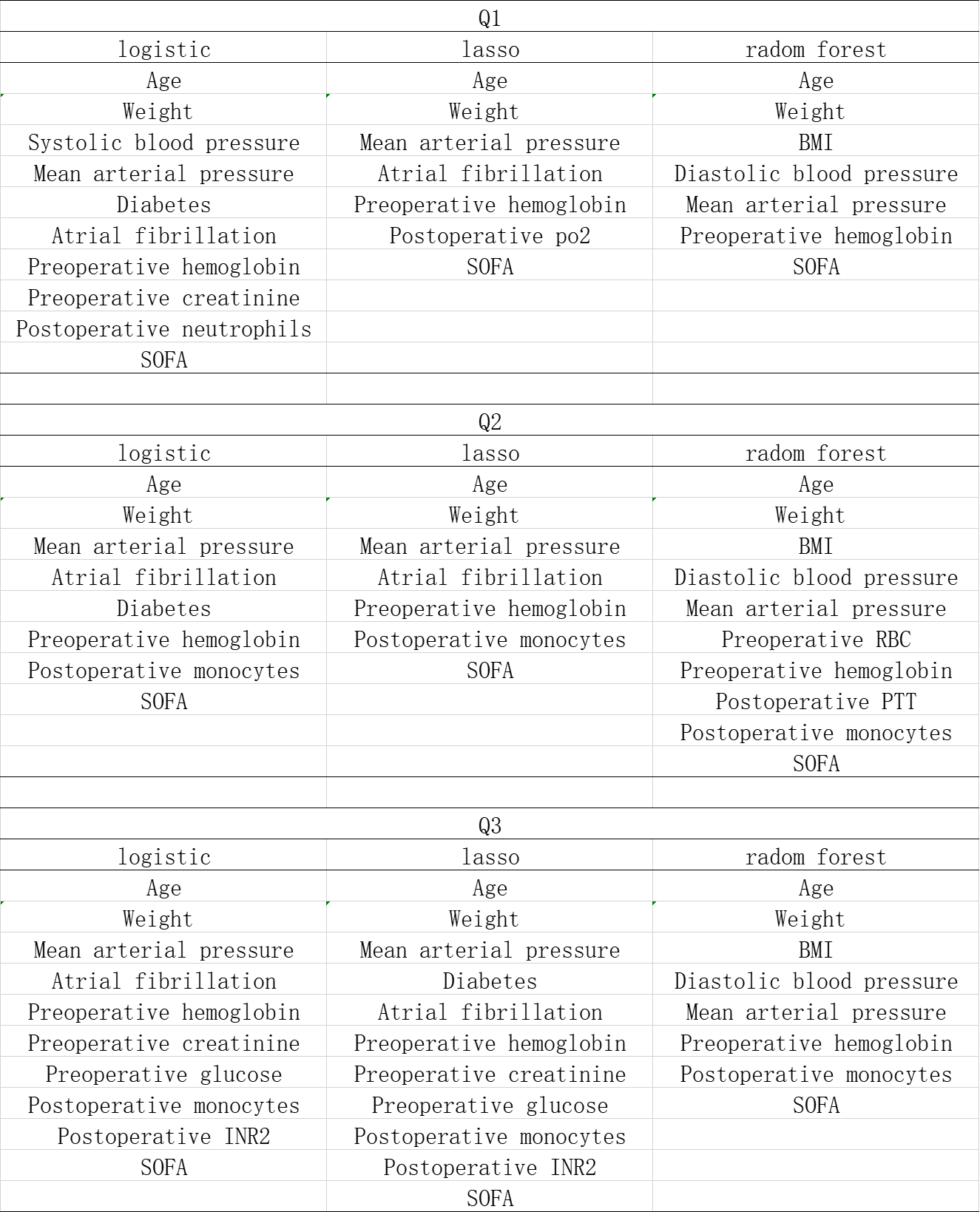

Supplement: S5 Table — (PNG) [file pone.0325151.s005.png]
